# Loss of Health Promoting Bacteria in the Gastrointestinal Microbiome of PICU Infants with Bronchiolitis: A Single-Center Feasibility Study

**DOI:** 10.3390/children9010114

**Published:** 2022-01-17

**Authors:** Madeleine M. Russell, Mara L. Leimanis-Laurens, Sihan Bu, Gigi A. Kinney, Shao Thing Teoh, Ruth-Anne L. McKee, Karen Ferguson, John W. Winters, Sophia Y. Lunt, Jeremy W. Prokop, Surender Rajasekaran, Sarah S. Comstock

**Affiliations:** 1 Department of Food Science and Human Nutrition, Michigan State University, Lansing, MI 48824, USA; russe513@msu.edu (M.M.R.); busihan@msu.edu (S.B.); kinneygi@msu.edu (G.A.K.); comsto37@msu.edu (S.S.C.); 2 Department of Pediatrics and Human Development, College of Human Medicine, Michigan State University, East Lansing, MI 48824, USA; johnw.winters@helendevoschildrens.org (J.W.W.); jeremy.prokop@hc.msu.edu (J.W.P.); surender.rajasekaran@spectrumhealth.org (S.R.); 3 Pediatric Intensive Care Unit, Helen DeVos Children’s Hospital, Grand Rapids, MI 49503, USA; ruth-anne.mckee@spectrumhealth.org (R.-A.L.M.); karen.ferguson@spectrumhealth.org (K.F.); 4 Department of Biochemistry and Molecular Biology, Michigan State University, East Lansing, MI 48824, USA; teohshao@msu.edu (S.T.T.); sophia@msu.edu (S.Y.L.); 5 Department of Chemical Engineering and Materials Science, Michigan State University, East Lansing, MI 48824, USA; 6 Department of Pharmacology and Toxicology, Michigan State University, East Lansing, MI 48824, USA; 7 Office of Research, Spectrum Health, Grand Rapids, MI 49503, USA

**Keywords:** critical care, pediatrics, diet, gut microbiome, respiratory syncytial virus, viral bronchiolitis, antibiotics

## Abstract

The feasibility of gastrointestinal (GI) microbiome work in a pediatric intensive care unit (PICU) to determine the GI microbiota composition of infants as compared to control infants from the same hospital was investigated. In a single-site observational study at an urban quaternary care children’s hospital in Western Michigan, subjects less than 6 months of age, admitted to the PICU with severe respiratory syncytial virus (RSV) bronchiolitis, were compared to similarly aged control subjects undergoing procedural sedation in the outpatient department. GI microbiome samples were collected at admission (*n* = 20) and 72 h (*n* = 19) or at time of sedation (*n* = 10). GI bacteria were analyzed by sequencing the V4 region of the 16S rRNA gene. Alpha and beta diversity were calculated. Mechanical ventilation was required for the majority (*n* = 14) of study patients, and antibiotics were given at baseline (*n* = 8) and 72 h (*n* = 9). Control subjects’ bacterial communities contained more *Porphyromonas*, and *Prevotella* (*p* = 0.004) than those of PICU infants. The ratio of *Prevotella* to *Bacteroides* was greater in the control than the RSV infants (mean ± SD—1.27 ± 0.85 vs. 0.61 ± 0.75: *p* = 0.03). Bacterial communities of PICU infants were less diverse than those of controls with a loss of potentially protective populations.

## 1. Introduction

The first 2 years of life are critical in the establishment of the human gastrointestinal (GI) microbiome, the composition of which has been increasingly associated with long-term health outcomes [1,2]. The gut microbiota exists in a mutualistic relationship with the host and its surroundings, and its diversity increases rapidly in the first months of life through exposure to microbes from the diet, home, and environment [3]. These changes in gut microbiota coincide with a time when the first exposures to respiratory viruses occur. The GI microbiota has been described as a driver of critical illness [4]. Critically ill adult patients develop intestinal dysbiosis and experience a loss of health-promoting bacteria in their intestinal microbiome during intensive care unit (ICU) admissions [5,6]. However, this phenomenon is poorly understood for infants in the pediatric ICU (PICU).

The state of the gut microbiome in critically ill infants experiencing viral respiratory illness needs to be explored further. The gut–lung axis is an emerging area of interest that highlights microbe-microbe and microbe-host interactions in the lung and the gut [7]. The gut–lung axis can potentially influence the composition of the GI microbiota of infants within the PICU experiencing lung inflammation [8]. It has been established that translocation of gut bacteria to the lung during respiratory illnesses occurs and can play a role in alveolar inflammation [8]. This relationship between the gut and the lung is bidirectional, with acute and chronic diseases of the lung causing changes to the composition of the gut microbiota [8]. There is a need to develop an understanding of how the external environment affects the gut–lung axis and eventually relate this to patient outcomes in viral respiratory illness.

Investigating the loss of health promoting bacteria within the gut microbiomes of infants in the PICU during specific illnesses could further understanding of the relationship between critical illness and protective microbiota [9]. Respiratory syncytial virus (RSV) is an extremely contagious disease that is the leading cause of respiratory tract infections during infancy [10]. There is currently little understanding of the gut microbiota composition of infants with RSV in the PICU. Through the use of blood transcriptomics, we have previously shown that members of this RSV cohort presented experienced an extensive inflammatory response [11]. Our focus here was to determine how severely RSV in hospitalized infants impacts the gut microbiota and how various exposures (antibiotics, diet) modulate the GI microbiota. The results will provide further insight into the role of critical illness on the acute patient GI microbiota diversity, suggesting longitudinal studies to track the impacts of the changes over the long term. 

## 2. Material and Methods

### 2.1. Study Population, Site and Sample Collection

Approval for this study was given by the Helen DeVos Children’s Hospital IRB committee (2107-049-SH/HDVCH; 28 March 2017), as has been previously described [11]. This was a combined cross-sectional (controls) and longitudinal (PICU) design in an urban quaternary care children’s hospital in Western Michigan. Patients less than 6 months of age (excluding premature births less than 37 weeks gestation) who were admitted to the PICU for bronchiolitis and were positive for RSV (via nasopharyngeal samples by rapid polymerase chain reaction (PCR); Respiratory Pathogen Panel FilmArray, BioFire Diagnostics, Salt Lake City, UT, USA) were consented. For study subjects (*n* = 20), samples were collected at two time points: within 24 h of admission to the PICU and just prior to intravenous catheter (IV) removal or discharge from the PICU/hospital (average 3–4 days after admission to PICU). Age-matched control subjects with no known lung disease were recruited from the hospital’s sedation program (*n* = 10). Sedation control infants were fasted for 8 h from solid food intake and 4 h from clear liquid intake before sampling. At each sampling time point, we collected a peri-anal (external to the rectum) swab. Due to difficulties extracting DNA from some swabs (from lack of sample abundance), not all collected samples could be sequenced. Thus, the final sample size for microbiome analyses was 9 control infants, 12 RSV infants at baseline, and 16 RSV infants at 72 h (Supplemental Figure S1). All specimens were initially stored at −20 °C prior to being transferred to a −80 °C freezer until processing. 

Patient populations in the PICU are already quite heterogenous. In order to reduce this heterogeneity, we chose to limit participation to those infants with a specific infection. RSV infection was chosen because among patients in our PICU that is the most common infection leading to bronchiolitis. 

### 2.2. Data Collection 

Data was collected and managed using Research Electronic Data Capture (REDCap) tools. Basic demographic variables were abstracted from the local electronic medical record (EMR). Antibiotic treatment at the time of sample collection was extracted from the EMR. Dietary history, and mode of feeding (nil per os (NPO), per os (PO), tube feeding (TF) ± lipids, total parenteral nutrition (TPN) ± lipids) was abstracted from the dietitian’s notes in the EMR. Percent calories and protein were calculated from the resting energy expenditure [12] and daily required intake [13] in the 24-h period prior to sample collection to represent the most recent nutritional intake. Diet intake data was then categorized: less than or equal to 33% of needs met, greater than 33% but less than 99% of needs met, or greater than or equal to 99% of nutritional needs met. Additionally, diet intake was categorized in the following method: formula (FF), BM (breast milk), not eating (NE—infants consuming under 33% of total calories regardless of diet), mixed diet of FF and BM (MD). Control infants were grouped separately.

### 2.3. DNA Extraction, 16S Library Preparation and Sequencing

DNA was extracted from peri-anal swab samples (Qiagen PowerSoil DNeasy kit), and 16S rRNA gene libraries were created; sequencing was conducted as previously described [14]. Taxonomic and phylogenetic data were assigned using the SILVA reference database (release 102). For those samples included in the final analysis, the average number of reads per control sample was 84,957 and 60,102 reads per RSV sample. Sequences were subsampled to 10,000 reads per sample. Samples from 19 RSV infants were sequenced, with 12 samples being taken from infants at baseline and 16 samples taken at 72 h. Nine infants had data for both timepoints. Nine control samples were sequenced.

### 2.4. Statistical Methods

Bacterial diversity was analyzed using the vegan package in R [15]. Alpha diversity is the measure of infant within-sample diversity, with Chao 1 measuring the presence or absence of bacteria, while Shannon measures bacterial abundance and evenness. The Chao 1 and Shannon indices of alpha diversity and the Sorenson and Bray–Curtis metrics of beta diversity are reported. Beta diversity describes the bacterial community dissimilarity between two communities. The two beta diversity indices, Sorenson and Bray–Curtis, measure the presence and absence of bacteria and bacterial abundances, respectively. Paired t-tests of Shannon and Chao 1 alpha diversity were performed on alpha diversity metrics for data across timepoints. For other comparisons, normality for alpha diversity was determined by using the Shapiro–Wilk test; differences between groups when data was normally distributed was tested by analysis of variance (ANOVA). For non-parametric data, Kruskal–Wallis was used to test differences between groups. Post hoc comparisons between groups were performed using Tukey’s HSD test. Beta diversity differences between groups were tested using permutational multivariable analysis of variance (PERMANOVA, adonis function) and differences in group variances were tested using PERMDISP (betadisper function) in the vegan package. Principal coordinate analysis (PCoA) plots were used to visualize bacterial community beta diversity. Benjamini–Hochberg was used to correct for a false discovery rate [16]. Individual taxa differences between groups were determined using a negative binomial model in the MASS package [17,18]. *P*-values less than 0.05 were considered significant. Ratios were compared using the Kruskal–Wallis test as implemented by the npar1way procedure in SAS version 9.4 (Cary, NC, USA). 

## 3. Results

Patient demographic, antibiotic use, mode of feeding, diet, and the percentage of calories and protein are summarized for both 24 and 72 h (Table 1). Most PICU infants (*n* = 15) had severe infection, which was defined clinically as requiring mechanical ventilation. The median age of study participants was 9 weeks. At baseline, twelve infant’s stool samples were successfully sequenced. Of those samples successfully sequenced from the baseline, nine RSV infants consumed infant formula, one consumed BM, one consumed MD, and one was NPO. For all PICU infants enrolled in the study, the most common mode of feeding at baseline was a nasogastric tube (*n* = 5), followed by PO or none at (*n* = 3) each, and one infant was fed by orogastric (OG) tube. 

At 72 h, for those infants whose stool samples were sequenced (*n* = 16), the majority of RSV infants were FF (*n* = 9). Four infants consumed MD, and three consumed BM. At 72 h, thirteen RSV infants were fed NG, with one fed NJ, one PO, and one OG. Six of the sedation-control infants were FF, with two consuming BM and two fed a MD. Additionally, at baseline, for those RSV infants whose microbiome samples could be sequenced, six were receiving antibiotics, while five were not, and one infant’s antibiotic intake could not be determined. With respect to antibiotic use at 72 h, for those RSV infants whose microbiome samples could be sequenced, seven infants were receiving antibiotics and nine infants were not receiving antibiotics. 

Details of all pharmacological agents including antibiotics, anticoagulants, corticosteroids, diuretics, electrolytes, gastric acid secretion reducers, treatments for hypokalemia, laxatives, neuromuscular blocking agents, opioids and sedatives administered to infants is now included in Supplemental Table S1. Opioids were the most frequently administered pharmacological agents with more than half of the patients receiving doses at any time point (fentanyl: 12 at baseline; 15 at 72 h). Cephalosporins were the most frequently administered antibiotic class (5 at baseline; 4 at 72 h), and penicillins were the second most frequent (3 at baseline; 5 at 72 h). The use of gastric acid suppression regimes (such as ranitidine), which has previously been reported as predominantly used in FF infants, was not reflected in this data set. 

Additional clinical data are reported in Supplemental Table S2, which reports that 13/20 (65%) patients had a central venous line at some point during their ICU stay. Co-infections were reported in 17/20 (85%) and included pneumonia due to *Streptococcus pneumoniae* (3; 15%), pneumonia due to *Hemophilus influenzae* (3; 15%), pneumonia due to other specified bacteria (3; 15%), pneumonia due to methicillin (penicillin) susceptible *Staphylococcus aureus* (2; 10%), influenza due to identified novel influenza A virus with pneumonia (1; 5%), sepsis of newborn due to *Escherichia coli* (1; 5%), pneumonia due to other aerobic Gram-negative bacteria (1; 5%), and others.

**Figure 1:**
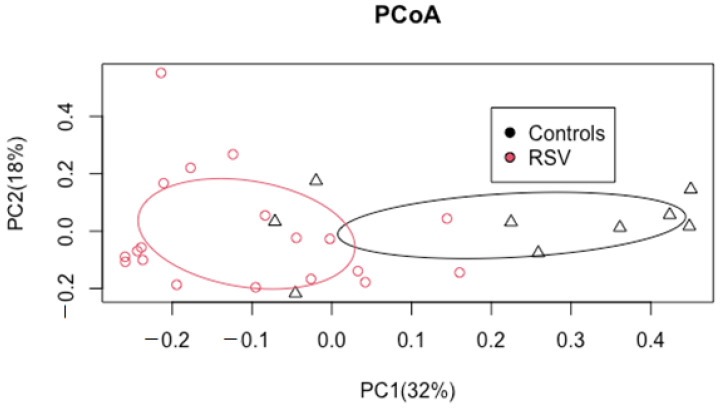
Gastrointestinal bacterial community dissimilarity based on the Bray–Curtis metric.

The diversity of the GI microbiota samples of infants with RSV was compared to the diversity of GI microbiota samples collected from control infants. In this analysis, data from a single sample was included for each infant. Most of the samples were from the 72-h time point (*n* = 16). Samples from three additional PICU patients were also included in the analysis, but these were samples from the 24 h time point, as these infants did not have sequencing data from samples collected at 72 h. Bacterial communities in the GI tract of the control infants had greater richness (more bacteria present) than those of the RSV infants (Table 3, Supplemental Figure S2, Supplemental Table S3). The Bray–Curtis beta diversity is plotted (Figure 1) using principal coordinates analysis (PcoA), where the beta diversity of the GI microbiotas of control and RSV infants separated on the PcoA axis 1. Bacterial communities of RSV infants were dissimilar to those of control infants. 

The microbiotas of the control infants are represented by black triangles. The microbiotas of infants with RSV are represented by red circles. Dots that are closer to each other indicate infants with a more similar gastrointestinal microbiota composition. Ellipses are centered on the median for the group and indicate one standard deviation from the median for the Bray–Curtis dissimilarity scores of the dots in each group. Control infants had GI microbiotas that were distinct from those of RSV infants (PERMANOVA, *p* = 0.0002; PERMDISP, *p* = 0.55).

Additional analyses compared the GI microbiota in the following ways: (1) at baseline and 72 h, (2) by disease severity, and (3) in antibiotic exposed versus those not exposed. Alpha diversity, measured by the Shannon and Chao 1 indices, was similar at baseline (*n* = 12) and 72 h (*n* = 16) (Supplemental Figure S3). At baseline, abundance and evenness of gut bacterial composition was similar for infants with moderate (*n* = 6) or severe (*n* = 22) RSV (Supplemental Figure S4). The alpha diversity of the gut microbiota for infants with RSV who were taking antibiotics (*n* = 13) was similar to that for infants with RSV who had no antibiotic exposure (*n* = 12) (Supplemental Figure S5, Shannon). Thus, time, disease severity, and antibiotic exposure were not associated with the GI microbiota in this study sample.

We next analyzed the impact of nutritional exposures on the GI microbiota of infants in the PICU with RSV. Infants were grouped by dietary intake: control, MD, BM, FF, and NE. Due to a limited sample size, BM (24 h: *n* = 0; 72 h: *n* = 2) and MD (24 h: *n* = 1; 72 h: *n* = 4) were excluded from analyses, leaving control, FF, and NE categories. Regardless of the time point, the GI microbiota of NE infants and control infants had similar alpha (Supplemental Figure S6) and beta diversity (Supplemental Figure S7). However, the GI microbiota of FF infants differed from that of control infants but not from NE infants. The FF infants at baseline and 72 h had lower alpha diversity than the control infants as well as altered bacterial communities (Supplemental Figures S6 and S7, Supplemental Table S4). The bacterial communities of the control infants and NE infants were similar to each other, but the bacterial communities of infants who were FF were dissimilar to the controls. 

## 4. Discussion

We describe the GI microbiome in PICU patients (<6 months of age) with RSV bronchiolitis diagnosed by nasopharyngeal polymerase chain reaction (PCR) testing. Similar to prior reports, the GI microbiomes of PICU infants were less diverse than those of the controls [19,20,21]. However, we identified a unique feature in the GI microbiota of patients with viral bronchiolitis. The GI bacterial communities of these infants were deficient in *Prevotella* leading to a decreased *Prevotella* to *Bacteroides* ratio in the GI microbiotas of these patients.

*Prevotella* has traditionally been established as a commensal indicative of a healthier gut microbiome, and its abundance is positively associated with a plant-based diet [22]. Conversely, *Bacteroides* has been associated with high-fat, high-protein diets, and thus the *Prevotella* to *Bacteroides* ratio has been an area of interest for understanding health outcomes. Herein, the lower *Prevotella* to *Bacteroides* ratio observed within the RSV infant GI bacterial community compared to that observed in the controls was entirely due to the loss of *Prevotella,* as the relative abundance of *Bacteroides* was comparable in both the patients and controls. This shift in predominant bacterial composition could be used as a possible indication of disease state for infants through incorporation of rapid multiplex PCR testing for these bacterial genera using peri-anal swabs from infants. 

Further, our observations related to *Prevotella* highlight the potential role of the gut–lung axis in outcomes for pediatric patients with infectious lung diseases. Gut microbiota and their metabolites can influence mucosal immunity which in turn has been shown to affect the immune responses at other mucosal sites, such as the lung [23]. Metabolites produced by protective members of the gut microbiota can prevent damage caused by inflammation and influence outcomes for patients with lung diseases [23,24]. The lung has also been shown to influence the microbial composition and inflammation of the gut [25]. The loss of *Prevotella* from the GI microbiome of infants with RSV may result from a mucosal immune response generated in the lower airways, where *Prevotella* are common residents, and depletion of *Prevotella* from the lung during instances of lung disease has been previously observed [26]. A strong immune response against *Prevotella* in the lung could stimulate a similar strong response against *Prevotella* in the GI tract due to the mucosal immune response. A more detailed analysis of the specific strains of *Prevotella* in the intestinal tracts and lungs of this patient population is required to test this hypothesis. 

The cytokine IL-18, epithelial immune biology, and the bacterial genera *Prevotella* have been shown to be highly associated with each other, such that an altered abundance of GI *Prevotella* results in steady state IL-18 deficiency and risk of epithelial inflammation, which can be rescued with IL-18 supplementation [27]. Although we observed differences in GI bacteria, including *Prevotella*, in these PICU patients, none of these taxa were observed to differ in the blood of RSV vs. control samples based on a PAXgene tube RNAseq and KRAKEN2 annotation of bacteria for the same patients [11]. Our work on this hospitalized RSV cohort using blood PAXgene tube RNAseq shows an extensive modulation of systemic inflammatory signals which include higher levels of 31 genes connected to IL-18 biology (*AIM2*, *ARG1*, *CARD17*, *CASP5*, *CCR1*, *CD163*, *CD274*, *FCGR1A*, *FCGR2A*, *HGF*, *HP*, *IL18R1*, *IL18RAP*, *IL1B*, *IL1R1*, *IL1R2*, *IL1RN*, *IRF7*, *MAPK14*, *MMP9*, *MX1*, *NAIP*, *NLRC4*, *PPARG*, *RETN*, *SOCS3*, *SPI1*, *TLR2*, *TLR5*, *TLR8*, *TNFSF13B*) and 5 genes lower (*CCR3*, *CD1C*, *CXCL8*, *KLRB1*, *KLRK1*) in RSV patients [11]. Thus, our results in this cohort of infants supports prior work describing the intimate connection of IL-18 biology to *Prevotella* abundance. However, direct associations between the GI microbiome and serologic inflammasome will need to be tested in the future as this patient population is too heterogeneous and of insufficient sample size for such associations to be tested. 

Fecal samples of critically ill infants and children have significantly altered microbiota relative to their healthy counterparts. These results are consistent with prior reports [19,20]. The loss of commensal bacterial taxa that provide resistance against pathogenic bacteria has been observed in children with critical illness residing in the PICU [19,20,28]. Decreased taxa level abundances of *Bacteroides* and *Bifidobacterium* [19,28], *Faecalibacterium* and *Ruminococcus* [20] establish that PICU patients have a depletion of commensals in their GI microbiomes. Additionally, an increase in potentially pathogenic bacteria in the gut microbiotas of patients in the PICU and NICU settings has been documented, including *Enterobacteriaceae* [19,28] and *Enterococcus* [20,28]. Herein, RSV infants had a higher relative abundance of *Bifidobacterium* compared to controls, which conflicts with the previously published data that demonstrated loss of *Bifidobacterium* within infants with severe illness. However, we did observe that infants with RSV had an increased abundance of *Enterobacteriaceae*, a potentially pathogenic bacteria in the gut, which was one of the previously observed pathogenic taxa to be increased in a similar patient population [19,28]. Infant age, mode of feeding, and the built environment of the PICU all contribute to the development of individual gut microbiomes. The taxa level differences in our study population compared to prior published observations imply that the depletion of commensals and enrichment of pathogens may differ by geographical location and/or specific differences in study site practices. In other words, the microbiota changes may be based on the commensals and pathogens present in the unique PICU environments and inhabitants of each study site. Taxa level changes during PICU stay may be site specific. A single study which makes a comparison between sites, using identical sample collection and processing methods, is needed to understand the details of commensal loss and pathogen enrichment within GI microbiomes of infants residing in PICU settings. 

One challenge faced early in the implementation of this study was the feasibility of conducting a GI microbiome analysis using infant fecal samples within a PICU. Hence, we sought to inform methodology for future PICU GI microbiome work. Stool samples are considered the gold standard for non-invasive characterization of the GI microbiome and was initially the intended sampling method for this study. However, infants under 3 months of age do not produce much fecal matter, and infants with critical illness who are not consuming large amounts of food and liquids orally produce even less fecal matter. Thus, collecting stool samples from diapers was not a feasible method to acquire sufficient biological material for microbiome analyses. For the majority of samples, we were able to acquire sufficient sample using peri-anal swabs. There has been previous debate over the efficacy of rectal sampling as an accurate representation of the gut microbiome. However, existing research supports the use of rectal swabs and asserts that the swabs are nearly as accurate as stool samples in characterizing the gut microbiome [29,30]. What is not clear from prior work is the specific area of sample collection. Herein, peri-anal swabbing of the area immediately exterior to the rectum was implemented to reduce trauma to the rectum during a time when infants could have increased risk of bleeding. Surgical samples, biopsies and luminal brushes are all proposed methods to characterize the gut microbiome more accurately, although each carries challenges of invasiveness and contamination [31]. One issue that arose with the use of swabs was a low yield of DNA during DNA isolation procedures. Thus, about 25% of collected samples could not be sequenced, and our sample size was diminished in size. Therefore, when using swabs, it is important to plan for a sample size up to 30% greater than power calculations may predict given that some of the samples may yield little DNA. Due to our already limited sample size being further reduced, the results were thus limited in their generalizability outside of this patient population. However, since sample collection from diapers is impractical and samples taken directly from the intestine require more invasive practices for infants already experiencing critical illness, peri-anal swabs proved an adequate solution. 

As our initial research question was related to the influence of the infant diet on the gut microbiota, we wished to minimize dietary heterogeneity among our participants. Up to 6 months of age, infants in the PICU are fed solely infant formula, human milk, or a mix of infant formula/human milk. Thus, by limiting our population to those infants under 6 months of age, we could reduce the heterogeneity in the diets of our participants. Even with this age restriction, we still faced too much heterogeneity in the feeding practices for each infant in the PICU to make meaningful comparisons in gut microbiota composition by infant dietary intake. This research question is important to answer as nutrition is closely tied to the development of the gut microbiota, with the first two years of life being critical in the establishment of a healthy gut flora [32]. The modifiable nature of nutrition and its influence on the gut microbiome makes it an attractive area of research. During a PICU admission, half of all infants will develop malnutrition, despite evidence that proper nutrition has been tied with improved patient outcomes [33,34]. Therefore, we were interested in whether overall caloric level or type of nutrition influenced patient outcomes within our own patient population. However, due to the final achieved sample size of our study and concerns over power (Supplemental Tables S1 and S2), the generalizability of our nutritional observations is limited. In the future, we plan to enroll a larger sample perhaps with a recruitment strategy focused on enrolling equivalent numbers of infants fed specific diet types to improve our ability to detect differences in the GI bacterial communities of critically ill infants based on their nutritional intake. 

Plasma and urinary metabolites from the participants were additionally profiled (and are currently being published in an adjacent manuscript) by liquid chromatography-tandem mass spectrometry (LC–MS/MS) and partial least squares (PLS) modeling was used to examine relationships between plasma and urinary metabolites and bacterial taxa datasets, but the model had very low robustness suggesting lack of consistent metabolite-bacterial taxa relationships across samples. 

The results of this research highlight the necessity of future work investigating the impact of various exposures on microbiome composition shifts in infants during periods of illness. This work further supports existing data reporting commensal loss and pathogen enrichment within critically ill patients and is novel due to observing this shift in microbiome composition within infant patients. Although we found no evidence of differences in microbiota by dietary exposures, the lack of *Prevotella* in infants with bronchiolitis indicates microbial substrates (such as human milk oligosaccharides) are potentially lacking in nutritional formulations for PICU infants. Additionally, the taxa level changes we observed, specifically in relative *Prevotella* abundance, potentially contribute to the expanding evidence for the influence of the gut–lung axis during respiratory illness. We expect that the feasibility described herein of utilizing peri-anal swabs for GI microbiome characterization within infant populations will inform future methodologies in PICU research. A limitation of the study design was difficulty discerning between the impact of strictly the PICU or disease state on the GI microbiome. Future analyses building on this work should incorporate matched infants within the PICU without respiratory illness to determine strictly the impact of this factor on GI microbiome composition. Additionally, due to the limited sample size of this study, it is important to note the preliminary nature of our findings and their subsequent role in laying the groundwork for future explorations in this area. Ultimately, we establish that the gut microbiome of critically ill infants is distinct from those of healthy infants, and further investigation is needed to elucidate the precise mechanisms that contribute to the discrepancy in their composition.

## 5. Conclusions

Peri-anal swab samples enabled characterization of the GI microbiome in PICU and control infants. Bacterial communities in samples collected from PICU infants are less diverse than those of control infants. Given our limited sample size, especially for some categories of exposures, further conclusions about the impact of diet or antibiotic use on the GI microbiota of PICU infants must wait. However, the observation of decreased *Prevotella* to *Bacteroides* ratios in the GI microbiotas of the PICU infants provides evidence that the gut–lung axis is an important area of further research in this patient population.

## Figures and Tables

**Table 1:** PICU patient characteristics at 24 h (baseline) and 72 h.

					24 h Timepoint						72 h Timepoint				
ID	Age (mo)	Sex	Admit Weight (kg)	Sequenced (Yes/No)	Abx ^a^ (Yes/No)	Mode of Feeding ^b^	Diet ^b^	% Calories ^b,c^	% Protein ^b,c^	Sequenced (Yes/No)	Abx ^a^ (Yes/No)	Mode of Feeding ^b^	Diet ^b^	% Calories ^b,c^	% Protein ^b,c^
1	2–4	M	5.8	No	No	N/A ^e^	MD	N/A	N/A	Yes	Yes	NG	MD	100	100
2	2–4	M	6.5	Yes	No	PO	FF	<33	<33	Yes	Yes	NG	FF	94	100
3	2–4	M	5.7	No	No	BF	BM	<33	<33	Yes	No	NG	BM	99	70
4	>4	F	8.5	Yes	Yes	NG	FF	74	67	No	Yes	PO/NG	FF	0	0
5	<2	F	3.8	No	No	NG	FF	20	24	Yes	Yes	NG	FF	95	100
6	<2	M	3.9	No	Yes	None	BM	0	0	Yes	No	NG	BM	1	1
7	<2	F	3.2	Yes	Yes	None	FF	0	0	No	No	NG	FF	44	63
8	2–4	F	6.3	No	No	BF	MD	<33	<33	No	Yes	None	MD	0	0
9	<2	F	2.9	Yes	Yes	NG	FF	67	79	Yes	No	NG	FF	100	100
10	2–4	F	2.3	Yes	No	NG	FF	43	65	Yes	Yes	NG	FF	100	100
11	2–4	F	2.8	Yes	No	NG	FF	40	93	Yes	No	NG	FF	99	100
12	<2	M	4.2	No	N/A	N/A	BM	N/A	N/A	Yes	No	NJ	MD	100	100
13	2–4	F	5.0 kg	Yes	No	OG	MDBM + Beneprotein	76	71	Yes	No	OG	MD	100	72
14	<2	F	2.7	Yes	Yes	NG	BM	10	10	Yes	Yes	NG	BM	77	80
15	2–4	F	4.9	Yes	No	PO	FF	13	14	Yes	No	PO	FF	77	84
16	<2	F	4.2	No	No	NG	MD: BM + Enf Inf	96	81	Yes	No	NG	FF	100	98
17	2–4	F	3.3	Yes	Yes	None	FF	0	0	Yes	Yes	NG	FF	97	100
18	<2	M	3.1	Yes	Yes	PO 85% NG 15%	FF	16	23	Yes	No	NG	FF	100	100
19 ^d^	2–4	F	6.0	No	Yes	NG	BM	32	17	Yes	Yes	NG	MD:BM + Beneprtein	100	100
20	2–4	M	6.4	Yes	No	NPO	NPO	0	0	No	N/A	N/A	N/A	N/A	N/A

^a^ Taking antibiotics at time of sample collection; details of antibiotic administration included in Supplemental Table S3; ^b^ Mode of feeding, diet, % calories & % protein = 24 h period prior to sample collection; ^c^ % calories and % protein estimated for baseline if <33 listed; otherwise calculated; ^d^ Pt 19, time 2: breast milk + protein modular (beneprotein powder); ^e^ N/A = information was not available. The ratio of *Prevotella* to *Bacteroides* was greater in control infants than in RSV patients (mean ± SD, 1.27 ± 0.85 vs. 0.61 ± 0.75: *p* = 0.03). Multiple taxa in the bacterial communities of infants with RSV in the PICU differed in abundance from those observed in the bacterial communities of control infants (Table 2). The GI microbiota of RSV and control infants differed with respect to seven of these highly abundant taxa. Microbiotas of RSV infants had greater abundance of *Bifidobacterium*, *Enterobacteriaceae*, *Lachnospiracaea intcertae sedis*, and *Enterococcus* compared to control infants. Control infants had greater abundance of *Prevotella*, *Clostridiales* and *Porphyromonas* compared to RSV infant bacterial communities.

**Table 2:** Relative abundance of bacterial genera in RSV and control infants at a single timepoint.

Taxa	Controls (Mean ± SD)	RSV (Mean ± SD)	*p*-Value ^a^
*Lachnospiraceae_unclassified*	1.9 ± 2.2	4.3 ± 3	0.071
*Bifidobacterium*	7.3 ± 9.4	20.8 ± 11.7	0.011
*Enterobacteriaceae_unclassified*	9.5 ± 7.2	23.7 ± 18.7	0.005
*Veillonella*	11.6 ± 5.9	10.9 ± 8.1	0.888
*Bacteroides*	10.1 ± 6.5	9.2 ± 11.2	0.888
*Prevotella*	10.9 ± 9.5	3.1 ± 4.9	0.019
*Clostridium_sensu_stricto*	8.3 ± 12.4	3.6 ± 4.6	0.079
*Clostridiales_unclassified*	2.4 ± 3.7	0.6 ± 1.5	0.019
*Clostridium_XlVa*	1.7 ± 1	1.1 ± 2.5	0.556
*Lachnospiracea_incertae_sedis*	2.1 ± 2.2	5.7 ± 4.3	0.025
*Porphyromonas*	4.6 ± 7.4	0.1 ± 0.3	0.015
*Enterococcus*	0.1 ± 0.1	1.4 ± 2.6	<0.001
*Megasphaera*	1.5 ± 1.7	1.1 ± 3.2	0.773
*Salmonella*	3 ± 3.2	3.1 ± 7.9	0.973

^a^ FDR corrected *p*-values. SD: standard deviation; RSV: respiratory syncytial virus.

**Table 3:** Alpha diversity of the gut microbiota for all infants at a single timepoint.

Measures	Controls	RSV	*p*-Value
	Median (min, max)	Median (min, max)	
Chao1	106.2 (84.2, 200.1)	87.3 (42.0, 189.0)	0.02
Shannon	2.8 (2.2, 3.2)	2.1 (0.8, 3.0)	0.001

RSV: respiratory syncytial virus.

## Data Availability

The data are not publicly available due to privacy concerns given the small sample size. However, the data presented in this study are available upon request from the corresponding author.

## Supplementary Materials

The following supporting information can be downloaded at: https://www.mdpi.com/article/10.3390/children9010114/s1, Figure S1: Flow diagram of patients in the study; Figure S2: Boxplots of (A) Chao1 indices (*p* = 0.02) and (B) Shannon indices (*p* = 0.001) for RSV (*n* = 19) versus control infants (*n* = 9); Figure S3: Boxplot of Chao1 index (*p* = 0.13) for infants at baseline (*n* = 12) and 72 h (*n* = 16) versus controls (*n* = 9). Figure S4: Boxplot of Chao1 index (*p* = 0.18) for PICU infants with moderate (*n* = 6) or severe (*n* = 22) RSV versus controls (*n* = 9). Figure S5: Boxplot of Shannon index (*p* = 0.29) for infants taking antibiotics (yes; *n* = 13) versus those not taking antibiotics (no; *n* = 12) at baseline. Figure S6: Boxplots of (A) Chao 1 indices (*p* = 0.15) and (B) Shannon indices (*p* = 0.02) for FF (*n* = 4), or NE (*n* = 7) infants vs controls (*n* = 9) at baseline. Figure S7: Gastrointestinal Bacterial Community Dissimilarity Based on the Bray–Curtis Metric; Table S1: Medications prescribed for RSV infants (*n* = 20). Table S2: Co-infections and Central Venous Catheters for RSV infants (*n* = 20). Table S3: Table of effect sizes and power analyses for alpha diversity in supplemental figures. Table S4: Table of effect sizes and power analyses for beta diversity.

## Abbreviations

Analysis of variance (ANOVA), breast milk (BM), formula fed (FF), gastrointestinal (GI), mixed diet (MD), consuming under 33% of total calories (NE), nasogastric (NG), nil per os (NPO), orogastric (OG), pediatric intensive care unit (PICU), per os (PO), principal coordinate analysis (PCoA), respiratory syncytial virus (RSV), tube feeding (TF), short chain fatty acids (SCFAs), 72 h (72 h).
