# Supplementary material for: Loss of Health Promoting Bacteria in the Gastrointestinal Microbiome of PICU Infants with Bronchiolitis: A Single-Center Feasibility Study
Source: Children (Basel). 2022 Jan 17;9(1):114. doi: 10.3390/children9010114 (PMC8774632; doi:10.3390/children9010114)
Supplement: Supplementary file 1 [file children-09-00114-s001.zip › children-1525290-supplementary.pdf]

Supplemental Figures and Tables

Supplemental Figure S1. Flow diagram of patients in the study. Nine RSV patients had samples at both 24 hrs and 72hrs (paired samples) that were sequenced.

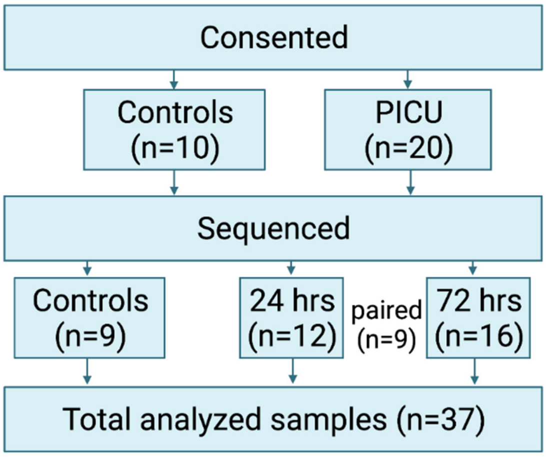

Supplemental Figure S2. Boxplots of A) Chao1 indices (p=0.02) and Shannon indices (p=0.001) for RSV (n=19) versus control infants (n=9). One sample was included for each RSV infant. Chao1 is an estimate of the number of species present in a community, also referred to as species richness. Shannon index considers the relative abundance of bacteria species to one another, also referred to as evenness and abundance.

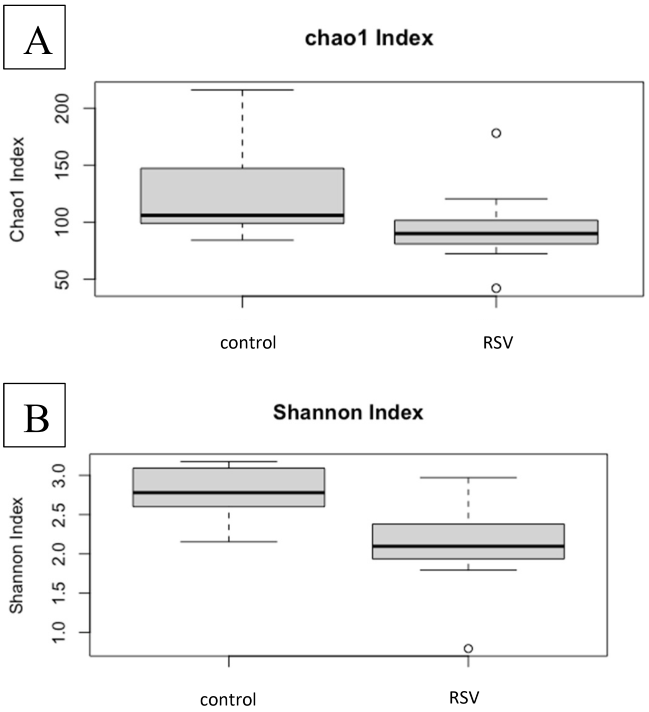

Supplemental Figure S3. Boxplot of Chao1 index (p=0.13) for infants at baseline (n=12) and 72 hr (n=16) versus controls (n=9).

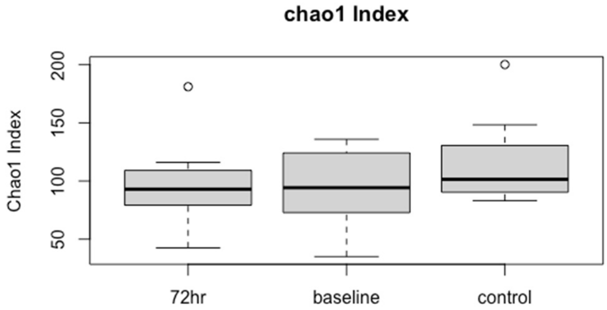

Supplemental Figure S4. Boxplot of Chao1 index (p=0.18) for PICU infants with moderate (n=6) or severe (n=22) RSV versus controls (n=9).

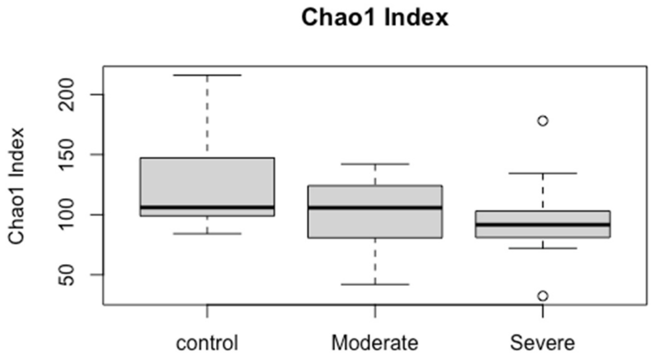

Supplemental Figure S5. Boxplot of Shannon index (p=0.29) for infants taking antibiotics (yes; n=13) versus those not taking antibiotics (no; n=12) at baseline.

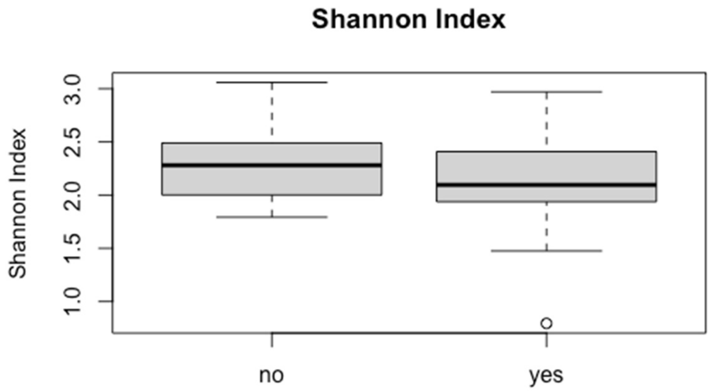

Supplemental Figure S6. Boxplots of A) Chao 1 indices (p=0.15) and B) Shannon indices (p=0.02) for FF (n=4), or NE (n=7) infants vs controls (n=9) at baseline. Boxplots C) Chao1 indices (p=0.17) and D) Shannon indices (p=0.01) for infants being FF (n=10), NE (n=1) vs. controls at 72 hours. The GI microbiota of FF infants but not from those of control infants.

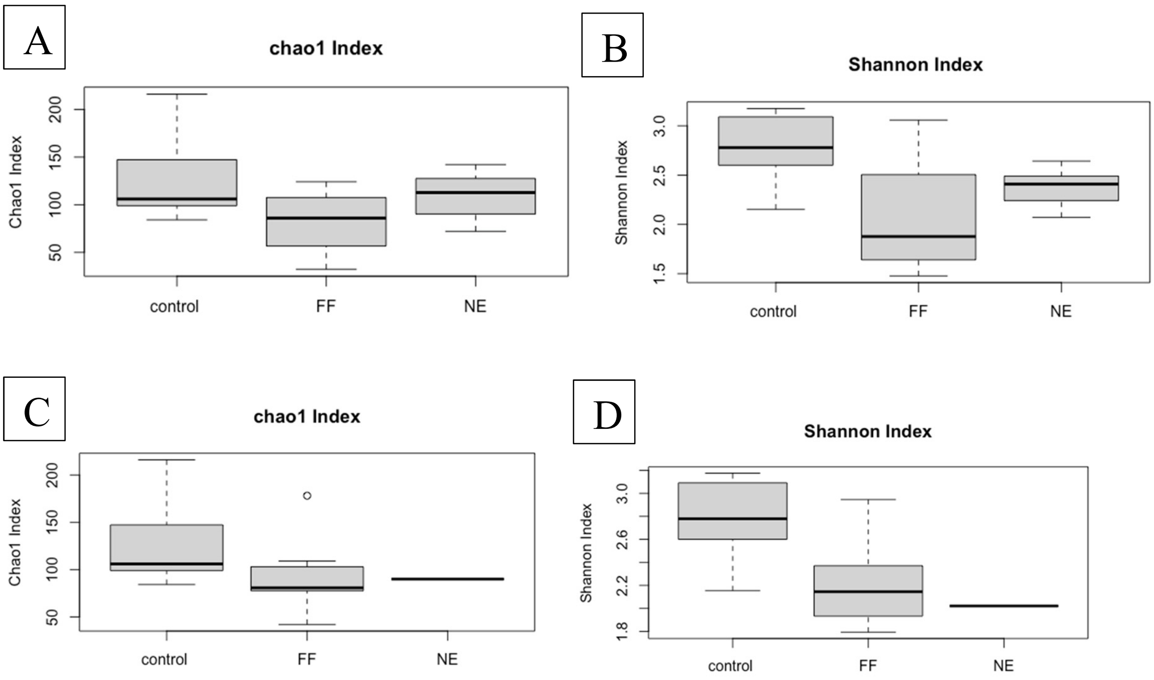

Supplemental Figure S7. Gastrointestinal Bacterial Community Dissimilarity Based on the Bray-Curtis Metric. A) Represents bacterial community dissimilarity based on type of feeding at baseline (permanova; p=0.005, permdisp; p=0.11) while B) is the bacterial dissimilarity at 72 hr (permanova; p=0.01, permdisp; p=0.03). The microbiota of the control infants are represented by black plus marks. The microbiota of FF infants are represented by red circles, and NE infants are represented by green triangles. Ellipses are centered on the median and indicate one standard deviation from the median for the Bray-Curtis dissimilarity scores of the dots in each group. FF infants had altered GI microbiota composition compared to control infants but not NE infants. NE infant bacterial communities and controls were similar.

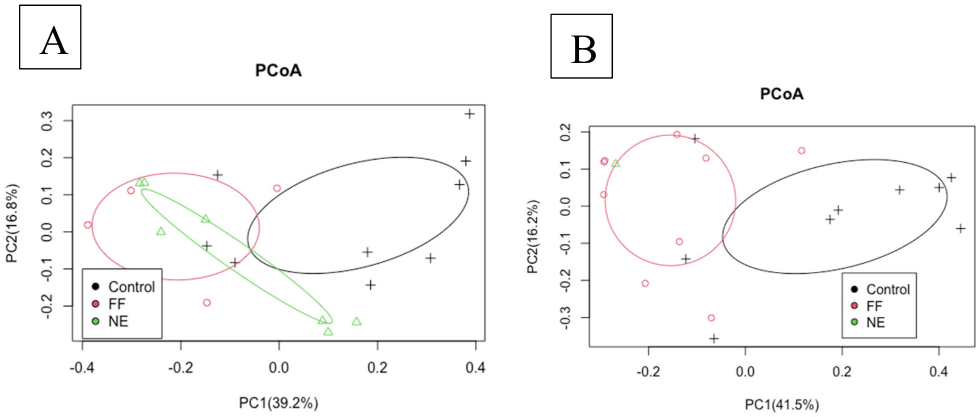

Supplemental Table S1. Medications prescribed for RSV infants (n=20)

Medication	Prior to Admit	Baseline	72 hrs
**Antibiotics**			
Cephalosporin			
Ceftazidime	0	1	1
Ceftriaxone	0	4	3
Lincosamide			
Clindamycin	0	0	1
Penicillin			
Amoxicillin	0	0	1
Ampicillin	0	3	4
**Anticoagulant**			
Heparin	0	2	2
**Corticosteroids**			
Hydrocortisone	1	0	1
Methylprednisolone	0	1	0
**Diuretic**			
Furosemide	0	5	3
**Electrolytes**			
Sodium Chloride	1	1	0
**Gastric Acid Secretion Reducers**			
Ranitidine	1	3	1
**Hypokalemia**			
Potassium Chloride	0	2	0
**Laxative**			
Glycerin suppository	0	0	1
Senna	0	1	0
**Neuromuscular blocking agents**			
Vecuronium	0	9	2
**Opioid**			
Dexmedetomidine	0	10	7
Fentanyl	0	12	15
**Sedative**			
Midazolam	0	9	4

Supplemental Table S2. Co-infections and Central Venous Catheters for RSV infants (n=20)

ID	Co-infection	Procedure	Other
1	Pneumonia due to Streptococcus pneumoniae	Percutaneous Central Venous Catheter (CVC)	N/A
2	Pneumonia due to Hemophilus influenzae	Percutaneous Central Venous Catheter (CVC)	Other viral agents as the cause of diseases classified elsewhere
3	Pneumonia due to Methicillin susceptible Staphylococcus aureus	Percutaneous Central Venous Catheter (CVC)	Streptococcal infection, unspecified site
4	Pneumonia, unspecified organism	None specified	N/A
5	Pneumonia due to Streptococcus pneumoniae	Percutaneous Central Venous Catheter (CVC)	N/A
6	Influenza due to identified novel influenza A virus with pneumonia	Percutaneous Central Venous Catheter (CVC)	N/A
7	Pneumonia due to other specified bacteria	Percutaneous Central Venous Catheter (CVC)	N/A
8	None	None specified	N/A
9	None	Percutaneous Central Venous Catheter (CVC)	N/A
10	Pneumonia due to Methicillin susceptible Staphylococcus aureus; Pneumonia due to Hemophilus influenzae	Percutaneous Central Venous Catheter (CVC)	Acute bronchiolitis due to other specified organisms; Enterovirus infection, unspecified
11	Enterovirus infection, unspecified	None specified	N/A
12	Pneumonia due to other specified bacteria	None specified	N/A
13	Pneumonia due to Methicillin susceptible Staphylococcus aureus	Percutaneous Central Venous Catheter (CVC)	N/A
14	Sepsis of newborn due to Escherichia coli	Percutaneous Central Venous Catheter (CVC)	N/A
15	Pneumonia, unspecified organism	None specified	N/A
16	Pneumonia due to Hemophilus influenzae	Percutaneous Central Venous Catheter (CVC)	Rhino enterovirus
17	Unspecified bacterial pneumonia	Percutaneous Central Venous Catheter (CVC)	N/A
18	Pneumonia due to Streptococcus pneumoniae	None specified	Other coronavirus as the cause of diseases classified elsewhere
19	Pneumonia due to other aerobic Gram-negative bacteria	Percutaneous Central Venous Catheter (CVC)	N/A
20	None	None specified	N/A

All patients were diagnosed with ICD10 Code Name of “Acute respiratory failure with hypoxia”.

Supplemental Table S3. Table of effect sizes and power analyses for alpha diversity in supplemental figures.

Figure	Effect Size	Power
1a[a]	0.36	46%
1b[b]	0.65	92%
2a	0.26	26%
2b	0.32	36%
3	0.19	16%
4	0.22	18%
5a	1.41	100%
5b	0.55	50%
5c	0.39	27%
5d	0.73	77%

[a,b]This data is also presented in Table 3 in the manuscript.

Supplemental Table S4. Table of effect sizes and power analyses for beta diversity.

Figure	Effect Size	Power
1	0.13	39%
Supplemental Figure 7a[a]	0.15	74%

[a]Due to uneven sample size in Fig 7b, no power calculation was completed.
